# Whole Body Vibration Triggers a Change in the Mutual Shaping State of Intestinal Microbiota and Body's Immunity

**DOI:** 10.3389/fbioe.2019.00377

**Published:** 2019-11-29

**Authors:** Ning Song, Xia Liu, Qiang Feng, Mengchen Xu, Xiang Lan, Meihui Li, Rutao Liu, Caixia Li, Tianyi Dong, Deqiang Wang, Shili Liu

**Affiliations:** ^1^School of Basic Medical Science, Shandong University, Jinan, China; ^2^Department of Rehabilitation Medicine, Binzhou Medical University Hospital, Binzhou, China; ^3^Shandong Provincial Key Laboratory of Oral Tissue Regeneration, Department of Human Microbiome, School and Hospital of Stomatology, Shandong University, Jinan, China; ^4^Shandong Engineering Laboratory for Dental Materials and Oral Tissue Regeneration, Department of Human Microbiome, School and Hospital of Stomatology, Shandong University, Jinan, China; ^5^State Key Laboratory of Microbial Technology, Shandong University, Qingdao, China; ^6^School of Environmental Science and Engineering, Shandong University, Jinan, China; ^7^Department of Evidence Identification, Institute of Forensic Science of China, Beijing, China; ^8^Department of Breast Thyroid Surgery, Shandong Provincial Hospital, Shandong University, Jinan, China

**Keywords:** whole body vibration, immune cell differentiation, Treg, mouse microbiota, human microbiota, correlation, physical characteristics, *Lactobacillus*

## Abstract

Whole body vibration (WBV) is a non-invasive physical therapy that has recently been included in the hospital's patient rehabilitation training catalog, but its health effects have not been sufficiently studied. In the present study, to examine the possible effects of WBV on immune cell differentiation, the IFN, IL-4,−17, F4/80 and CD3,−4,−8,−11b,−11c,−19 markers were used to characterizing the cells in mouse spleen. The results showed that the CD4 and CD25 positive lymphocytes in the spleen were significantly increased in the WBV group, and the population of Treg cells was enhanced significantly in response to WBV. Since the differentiation in immune cells is usually associated with microbiota, therefore the intestinal flora was characterized in mice and human individuals. The results indicated that WBV significantly reduced the α-diversity of mouse intestinal microbiota. Moreover, the principal coordinate analysis (PCoA) results indicated that the β-diversities of both mice and human fecal microbiota increased after WBV. Analysis of the bacterial composition indicated that the contents of a variety of bacteria changed in mice upon the stimulation of vibration, such as *Lactobacillus animalis* in mice, and *Lactobacillus paraplantarum* and *Lactobacillus sanfranciscensis* in human. The succeeding correlation analysis revealed that some bacteria with significant content variations were correlated to the regulatory T cell differentiation in mice and physical characteristics in human. Our research will provide the basis for future non-invasive treatment of microbial and immune related diseases.

## Introduction

Non-invasive therapy (NIT) refers to a therapeutic strategy that the body is not invaded or cut open during medical treatment. The risks associated with surgery, financial costs and lengthy body recovery time have led to the development of many non-invasive technologies (Kennedy et al., 2015). Whole body vibration (WBV) is a NIT method, which acts like a mild exercise on the body (Godinez et al., 2014). WBV is a common training method for improving athletic performance and maintaining astronauts' skeletal muscle mass and strength, it has recently been included in the hospital's patient rehabilitation training catalog. WBV machines usually provide rotational or vertical vibration stimulation, where vertical vibration is more commonly used for rehabilitation. Vertical vibration produces an upward thrust on the human body (Abercromby et al., 2007), which alternates with gravity to produce a fast, up and down shock that acts on the body's bones, muscles and nerves. Thereby WBV is a stimulus to the entire body, not to a specific local muscle group (Merriman and Jackson, 2009).

A number of literatures have been published on the effects of WBV on muscle strength and performance (Osawa et al., 2013; Rogan et al., 2015). The most basic function of vertical vibration is to stimulate the contraction of a large number of muscle fibers in a short period of time, which helps increase muscle strength, balance and muscle power (Machado et al., 2010; Sitja-Rabert et al., 2012), enhance mobility (Torvinen et al., 2002), reduce chronic pain (Rittweger et al., 2002), and stimulate limb blood circulation (Cochrane, 2011). Moreover, WBV may also affect other physiological systems of the body. Maddalozzo et al. reported that mice of the vibration group had significantly lower body fat than the control group (Maddalozzo et al., 2008). At the same time, bone mineral contents and bone density had been proved to increase significantly after a certain period of vertical vibration; the internal mechanism was the acceleration of bone formation and metabolism after using vertical vibration (Bleeker et al., 2005). Additionally, Boyle et al. reported that WBV combined with exercise reduced the possible danger of thrombosis or infarct greater than simple exercise, implying its possible role in the prevention and treatment of cardiovascular disease (Boyle and Nagelkirk, 2010). Regarding the effect on diabetes, Liu et al. reported that WBV reduced oxidative stress to ameliorate liver steatosis and improved insulin resistance in db/db mice (Liu et al., 2016). The research work of Reijne et al. supported this conclusion, and their results from mice demonstrated that WBV training tended to increase blood glucose turnover rates and stimulated hepatic glycogen utilization during fasting irrespective of age (Reijne et al., 2016). Also, Park et al. and Sa-Caputo et al. showed us the effects of WBV on pulmonary rehabilitation in patients with chronic obstructive pulmonary disease and improvement of local vasodilation in the microcirculation by the stimulation of endothelium (Park et al., 2015; Sa-Caputo et al., 2016).

However, other effects of the vibration stimulus need to be examined, and the mechanism responsible for the effect of this new intervention has not been clearly identified. Microbes that live on and in the human body, such as the oral cavity, gastrointestinal tract, and urinary tract, have been recognized as the key to understand our various abnormalities (Sekirov et al., 2010; Qin et al., 2012). Chronic inflammation is the driver of many diseases, and studies have shown that intestinal microbiota is closely related to the body's immune response and the occurrence of disease (Noverr and Huffnagle, 2004). The crucial characteristics of microbiota that affect immune response include biogeographical distribution, composition, and metabolites, etc. (Blander et al., 2017). Unfortunately, no one has yet studied the effects of WBV on the body's intestinal flora and immunity. In the present study, the effects of WBV on the immune cell differentiation and the composition of intestinal microbiota were investigated. The vibration used in the experiment was sonic vibrations. This vibration mode is adopted by most hospitals, and the vibration toward the patient is softer and less dangerous than mechanical vibration. Our results suggested that WBV may affect the immune cell differentiation by stimulating changes in intestinal bacterial composition. Our research will provide the basis for future non-invasive treatment of microbial and immune related diseases.

## Materials and Methods

### Treatments Related to Mouse and Human Volunteers

The 6-week old C57BL/6 mice in the same cage were divided into two groups: Treatment and Control. The mice in the treatment group were subjected to vertical vibration for 30 min per day for 30 days (frequency at 13 and intensity at 10 for 10 min; and then frequency at 17 and intensity at 20 for 20 min) on a vibrating instrument (Weibutexun, Jinan, China); the mice in the Treatment group were in a state of visible body vibration, but they were quiet and did not panic. After a period of vibration, the intestinal microbiota of the control and treatment groups were analyzed by sequencing. All animal studies were reviewed and approved by the ethic committee of School of Basic Medical Science, Shandong University (Jinan, Shandong Province, China). The human volunteers enrolled in the experiment were all from Binzhou Medical College, China. The volunteers were all healthy people, and their basic information is illustrated in Supplementary Table 1. Volunteers did not receive antibiotics, chemotherapy or radiotherapy for the first 3 months before the start of the experiment, nor did they consume probiotics. Throughout the experiment, volunteers every day performed a standing body vibration for 10 min (frequency 21 HZ, intensity 40) and then sat for vibration 10 min at the same frequency and intensity. All human studies were reviewed and approved by the ethic committee of Binzhou Medical University Hospital (Binzhou, Shandong Province, China).

### 16S Sequencing

Total fecal genome DNA was extracted with CTAB/SDS method, concentration and purity of the extracted genome DNA was checked by agarose gels electrophoresis. DNA was diluted with sterile water to the concentration of 1 ng/μl. Using about 10 ng of this template DNA and 0.2 μM of primers that had adapters and barcodes, the variable V3–V4 region of the 16S rRNA gene was amplified by PCR (25 PCR cycles) with Phusion® High-Fidelity PCR Master Mix (New England Biolabs). Subsequently, amplicons were sequenced on a 454 GS-FLX system (Roche, Mannheim, Germany).

According to the unique barcodes, the Paired-end reads were assigned to each sample, and FLASH was used to merge the Paired-end reads. R software package (Quantitative Insights Into Microbial Ecology) was employed for analysis of the sequences, and alpha- (within samples) and beta- (among samples) diversity were produced by in-house Perl scripts. In detail, reads were quality filtered by QIIME, and pick_*de_novo*_otus.py was used to make operational taxonomic units (OTUs) table. Sequences with ≥97% similarity were classified to an OTU. Then the representative sequence of an OTU was picked and taxonomic information was annotated using the RDP classifier. To obtain Alpha Diversity, the OTU table was rarified into three metrics: Chao1 represents the species abundance; the number of unique OTUs in each sample was represented by Observed Species and Shannon index. Based on these three metrics, Rarefaction curves were generated. The beta-diversity indexes, weighted and unweighted unifrac, were generated with QIIME.

### Flow Cytometry Analysis of Immune Cells

The spleens of the mice were collected, washed with phosphate Buffered Saline (PBS), and placed on ice in a 50 ml centrifuge tube containing PBS. Then the spleens were thoroughly ground with a copper mesh, and placed in a 50 ml centrifuge tubes, the volume was adjusted to 20–30 ml. After centrifuged at 1,200 rpm for 8 min, the supernatants were discarded, and 10 ml of red blood cell lysate was added to each tube, vortex, and place on ice for 7–8 min. Then centrifuged at 1,200 rpm for 8 min and the supernatant discarded once again, 2.88 ml percol, 0.32 ml of 8.5% NaCl solution, and 4.8 ml of PBS were added. The solution was transferred to a 10 ml centrifuge tube, and centrifuged at 2,000 rpm for 20 min; after the supernatant was discarded, 5 ml of PBS was added and the cells in the solution were counted. The number of cells required per well was estimated and pipette into a centrifuge tube. The cells were subsequently centrifuged at 1,200 rpm for 8 min, re-suspending in 1,640 and 10% FBS medium, and the stimulant PMA 30–50 μg/ml, ionomycin 1 μg/ml was added to the medium; after incubation in a 24-well plate at 1 ml/well for 1 h, 1×BFA blocker was added to reduce the extracellular release of cytokines. The cells were harvested within 4 h and placed in a flow tube, then washed with PBS and centrifuged at 1,200 rpm for 8 min; after the supernatant was discarded, the tube was blotted dry with absorbent paper, and 100 μl of PBS was added to each tube.

### Surface Antigen Labeling

Add 10 μl of antibody + PBS mix to each tube, repeatedly pipette and vortex to mix. The mix was wrapped with foil paper and placed in the dark at 4°C. Half an hour later, 2 ml of PBS was added and centrifuged at 1,200 rpm for 8 min. The supernatant was discarded, re-suspended in 300 μl of 1% poly-formaldehyde, and stored at 4°C.

### Intracellular Antigen Labeling

Vortex the cell solution and place at 4°C for 30 min, 1×permeability buffer 1 ml was added to each tube; after centrifugation at 1,200 rpm for 8 min, the supernatant was discarded, and 100 μl of membrane solution was added; vortex, placed at 4°C for 10 min and 10 μl labeled antibody + permeability buffer mix were added. After repeated pipette, the solution was placed at 4°C for 1 h in the dark, 2 ml PBS was then add and centrifuged at 1,200 rpm for 8 min. After discarding the supernatant, the cells were re-suspended with 300 μl PBS or poly-formaldehyde, and store at 4°C for flow cytometry.

### Statistical Data Analysis

The data were represented as mean ± standard deviation (SD), and each experiment had triplicate data. The Student's *t*-tests was used to calculate the *p*-value between the groups, and *p* < 0.05 (^*^) was considered as statistically significant.

## Result

### WBV Did Not Cause Any Movement Disorder or Other Changes in Behavior

**Figure 1:**
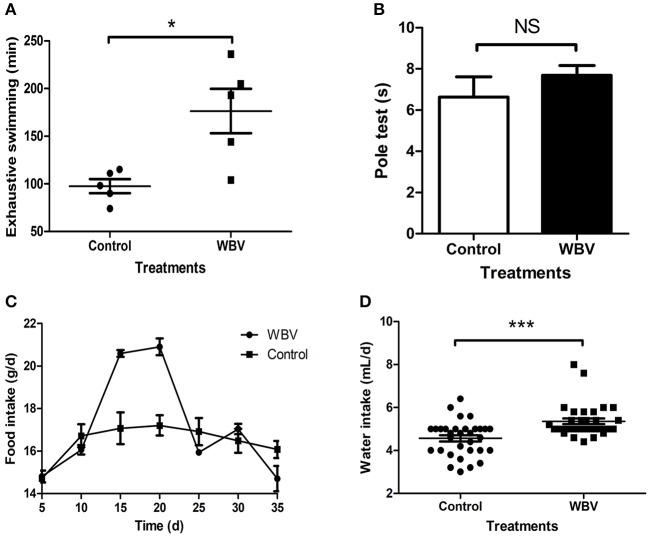
WBV did not cause any movement disorder or other changes in behavior. The Exhaustive swimming **(A)** results exhibited a significant difference between the mice with vibration or not, but the Pole test **(B)** results showed no difference; **(C,D)** the food and water intake of the mice. NS, not significant; *p < 0.05; ***p < 0.001.

The vibration instrument used in the experiment was shown in Supplementary Figure 1; it can provide whole body vibration, forcing the body muscles to be in a state of passive exercise. To explore the possible effect of WBV on physiological function of the body, the mice were placed on the vibration instrument for 30 min vibration every day for 35 days, and their dietary consumption and body weight changes were recorded during this process, as well as behavioral characteristics were assessed at the end. The exhaustive swimming result showed that the longest swimming time of the vibration group was significantly increased, almost twice the time of the control group (Figure 1A); proving that WBV can increase muscle strength and endurance. Moreover, the pole test result also showed no significant difference in the time of climbing pole between the two groups (Figure 1B, *p* = 0.36), illustrating that WBV treatment did not cause any movement disorder. The food intake of the WBV group mice increased on days 15, and peaked on the 20th day, but returned to similar to the control group level at the 25th day (Figure 1C). However, the average daily food intake of the WBV group mice during the entire period was not significantly different from the control group (Supplementary Figure 2). In contrast to food intake, there was a significant difference in water intake between the WBV group and the control group (Figure 1D), and the two groups both exhibited a certain degree of fluctuations in the amount of water consumed per day (Supplementary Figure 3). Additionally, the body weight results showed that the mice did not undergo significant changes in body weight after WBV (Supplementary Figure 4). The above results demonstrated that no changes in behavior of the mice were observed during the entire vibration experimental process.

### WBV Alters Regulatory T Cell Differentiation

**Figure 2:**
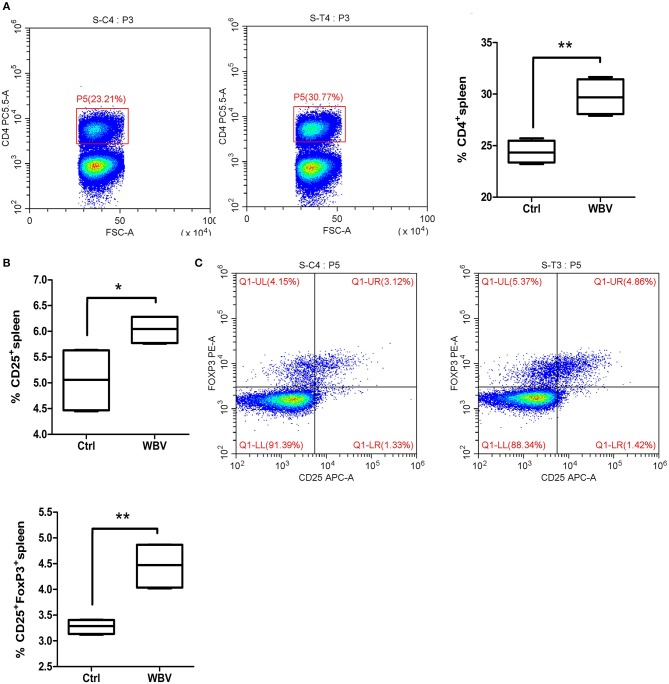
WBV alters regulatory T cell differentiation. Spleen CD4 **(A)** and CD25 **(B)** positive lymphocytes in the WBV group were significantly increased. **(C)** The population of regulatory T cells in the spleen were enhanced significantly. *p < 0.05; **p < 0.01.

To examine the effects of WBV on immunological development, we examined CD3,−4,−8,−11b,−11c,−19, and IFN, IL-4,−17, F4/80 markers of cells in the mouse spleen. The results showed that the CD4 (Figure 2A) and CD25 (Figure 2B) positive lymphocytes in the WBV group were significantly increased. As a result, the population of CD4^+^CD25^+^FOXP3^+^ Treg cells in the spleen was enhanced significantly (Figure 2C, *p* < 0.01). The above results indicate WBV alters regulatory T cell differentiation.

### WBV Altered the Composition of the Mouse Intestinal Microbiota

Since the differentiation in immune cells in the body are usually associated with changes in the human microbiota, therefore the possible change of microbiota after WBV treatment was characterized in mice. Fecal samples of the treatment group (ST) and control group (SC) were collected and their 16S rDNA amplicons were sequenced. After quality filtering, more than 0.56 million effective tags were harvested corresponding to a mean of 56,378 effective tags and 379 OTUs per sample (Supplementary Table 2 and Supplementary Figure 5). On average, the fecal samples from treatment group had similar number of effective reads (57,384) as the control group samples (55,372, *P* = 0.56). Similarly, there was no significant difference in the OTUs identified between the two groups of samples (*p* = 0.45), although the average of the fecal microbiota in the treatment group (398) was greater than that of the control group (361, Supplementary Figure 5). Furthermore, the Rarefaction curve tended to be saturated among all samples, suggesting that the OTUs have covered most of the bacterial species that exist whether vertical vibration or not (Supplementary Figure 6).

**Figure 3:**
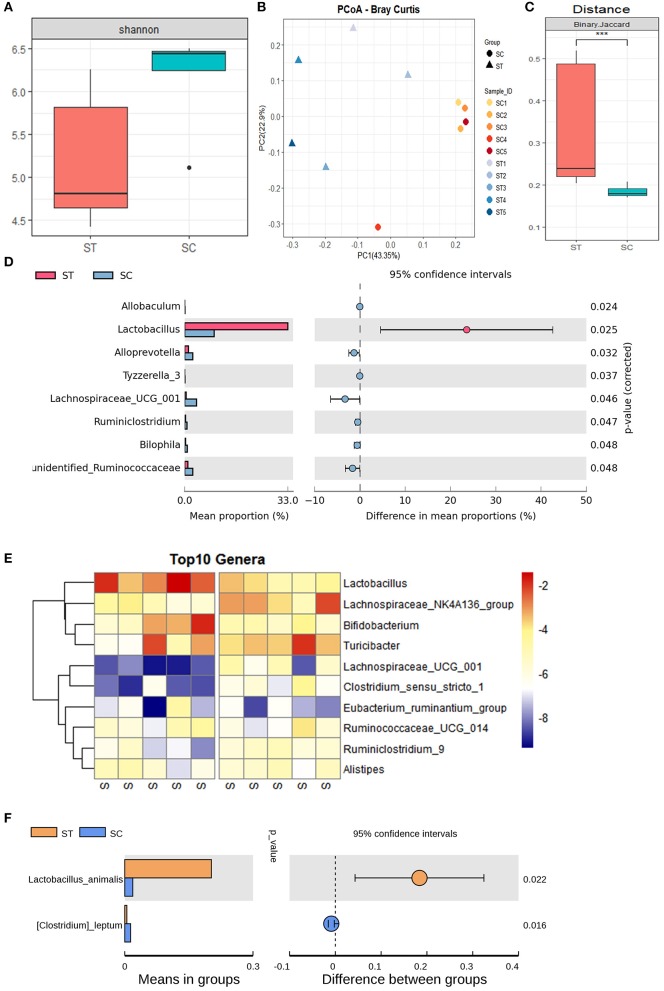
WBV altered the composition of the mouse intestinal microbiota. **(A)** The α-diversity of mouse intestinal microbiota; the PCoA **(B)** and distance of β-diversity **(C)** analysis of the mice in the vibration and control groups; **(D)** the bacteria with significant differences between the two groups; **(E)** the Heat map of the bacteria with significant differences; **(F)** the content of *Lactobacillus animalis* in the vibration and control groups. ***p < 0.001.

The α-diversity of mouse intestinal microbiota indicated that WBV significantly reduced the α-diversity of the sample (Figure 3A). In contrast, the principal coordinate analysis (PCoA) showed that the distribution of the treatment group samples was more scattered relative to that of the control group (Figures 3B,C), indicating that the β-diversity of the fecal microbiota increased after WBV (Figure 3C). A clustering analysis was then performed to quantify the degree of similarity of all the samples, and the result demonstrated that the control group samples were clustered together with a smaller Euclidean distance, while the WBV group samples were grouped in a larger Euclindean distance (Supplementary Figure 7). The results suggest that WBV affects the murine intestinal microbiome and makes the difference between individuals greater.

Then the intestinal microbial composition of the treatment and control mice were analyzed at the phylum, genus, and species levels, respectively. At the phylum level, the total amount of Firmicutes, Bacteroidetes, and Actinobacteria account for more than 90% of all fecal bacteria; meanwhile, the proportion of the most abundant phylum Firmicutes exceeded 45% of the total bacteria (Supplementary Figure 8). The observed effect of the vibration was the increased contents and variation between individuals in phylum Actinobacteria (Supplementary Figure 8). At the generic level, the genus Lactobacillus belonging to phylum Firmicutes, was the most abundant genus in the WBV group, covering approximately 30% of the entire bacterial mass (Supplementary Figure 9). However, its content in the control group was lower than Turicibacter and Lachnospiraceae_UCG_014, accounting for only about 5% of the total bacteria, and its content was significantly different between the two groups (Figures 3D,E). As a result, the vibration increases the content of *Lactobacillus animalis* in the intestine by more than 5 times at the species level (Figure 3F). In addition, WBV also triggered some other genera with high abundance, such as Lachnospiraceae_UCG_014 and Bifidobacterium, to change their contents in intestinal microbiota (Figure 3E).

### WBV Also Affects the Composition of the Human Intestinal Microbiota and Changes the Content of *Lactobacillus* spp.

**Figure 4:**
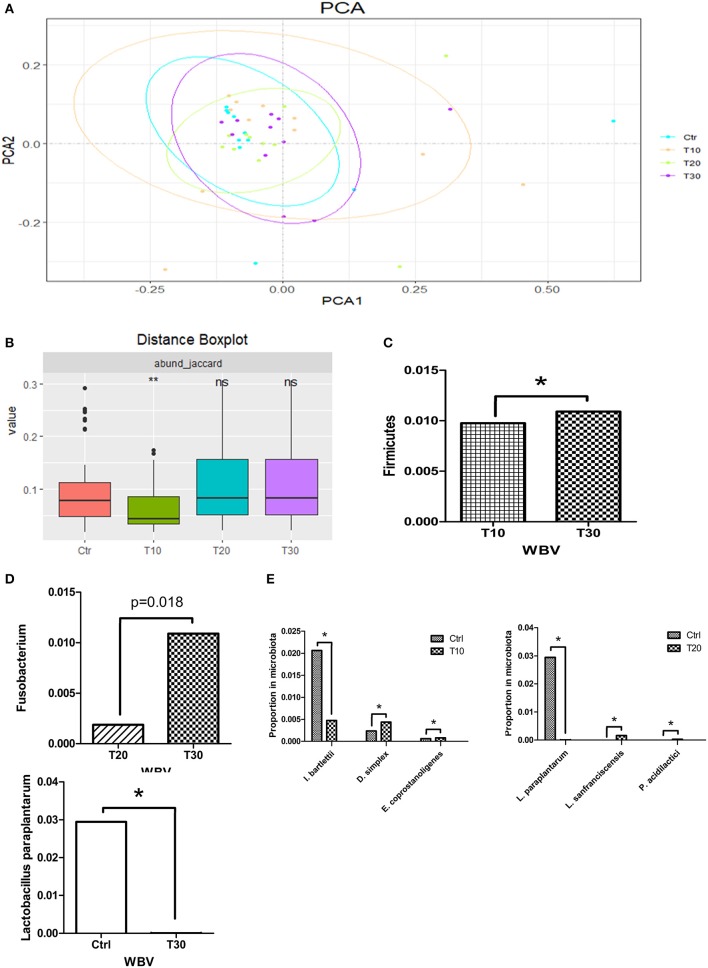
WBV also affects the composition of the human intestinal microbiota and changes the content of *Lactobacillus* spp. **(A)** PCA result of the human intestinal microbiota; **(B)** β diversity distance of the human fecal microbiota; phyla **(C)**, genera **(D)**, and species **(E)** with significant variation concomitant with longer time of vibration. *p < 0.05; **p < 0.01.

To study the effects of WBV on the human intestinal microbiota, we conducted 30-day vibration training on 11 volunteers, and the fecal samples of the volunteer were collected at 0 (control), 10 (T10), 20 (T20), and 30 (T30) days after WBV and sequenced. The Rarefaction curve tended to be saturated among all samples, suggesting that the OTUs have covered most of the bacterial species that exist in the feces (Supplementary Figure 10). Different from the results of mouse experiments, the α-diversities of the fecal microbiota of the WBV groups were not significantly different from that of the control group (Supplementary Figure 11). Unlikely, the principal components analysis (PCA) results showed that the microbial distribution of the T10 group was more scattered relative to those of the control and the other vibration groups (Figure 4A), suggesting that the β diversity of the fecal microbiota increases after short-term of WBV, but returns to the original state as the vibration time increases (Figure 4B), which is consistent with the results of the mouse experiment.

Then the intestinal microbial structures of the treatment and control groups were analyzed at the phylum, genus and species levels, respectively. At the phylum level, Firmicutes was the only phylum that had a difference in its content caused by WBV (Figure 4C). At the generic level, the content of genus Fusobacterium was significantly increased solely with longer time of vibration (Figure 4D and Supplementary Figure 12). At the species level, *Intestinibacter bartlettii, Desulfovibrio simplex*, and *Eubacterium coprostanoligenes* varied their contents in the feces with 10 days' vibration in comparison to the control; and the differentiated bacteria changed to be *Lactobacillus paraplantarum, Lactobacillus sanfranciscensis*, and *Pediococcus acidilactici* at the 20 days time-point; finally only *Lactobacillus paraplantarum* maintain significant difference in content after 30 days (Figure 4E). The results from the mice and human samples indicate that the WBV can change the composition of the intestinal microbiota of mice and humans, with a significant change in the proportion of certain bacteria such as *Lactobacillus* spp.

### WBV Induced Changes in Intestinal Microbiota Composition Were Correlated to the Regulatory T Cell Differentiation in Mice and Physical Characteristics in Human

**Figure 5:**
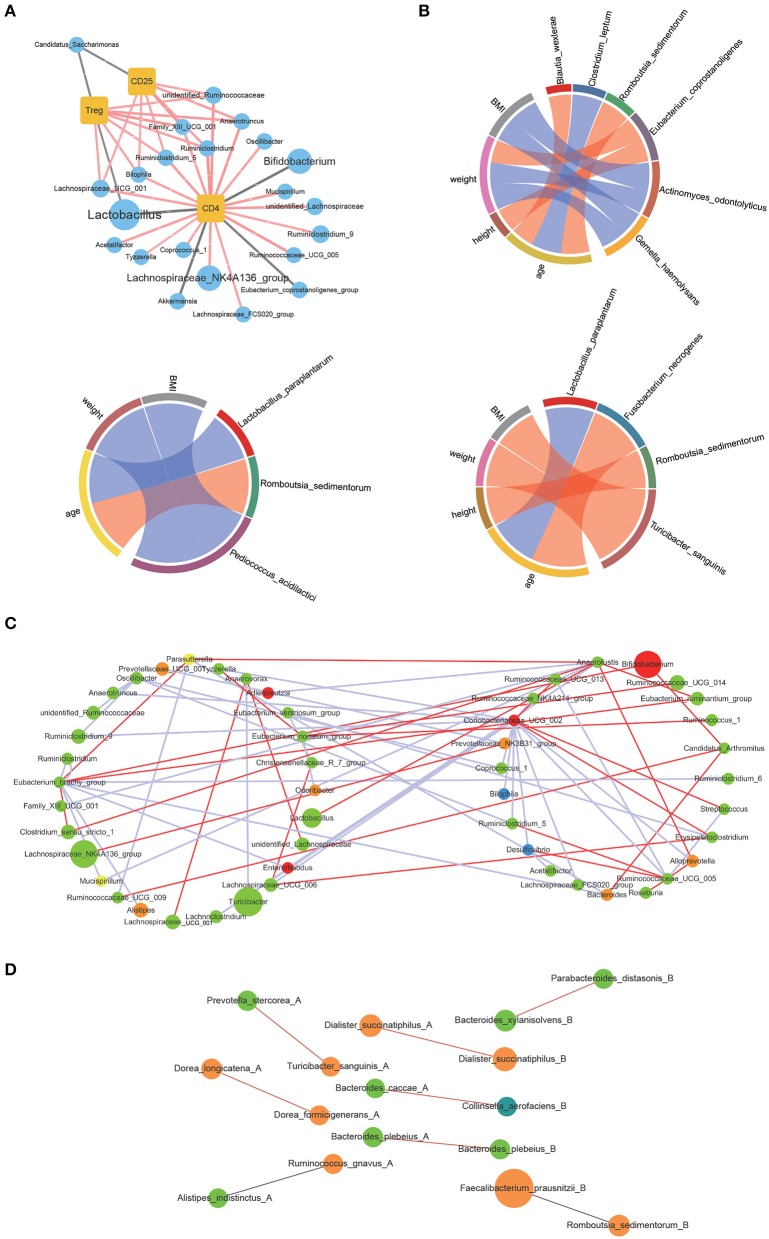
WBV induced changes in intestinal microbiota composition were correlated to the regulatory T cell differentiation in mice and physical characteristics in human. **(A)** Correlations of the CD4 and CD25 positive lymphocytes as well as Tregs with the intestinal bacteria; **(B)** correlations of the intestinal bacteria with clinical characteristics; the correlation network of mice **(C)** and human **(D)** fecal microbiota.

The correlations between the bacterial genus with significant differences in contents before and after WBV and the amount of CD4 and CD25 positive lymphocytes as well as Tregs were investigated by Spearman correlation analysis (“Hmisc” in R package). The results showed that CD4 positive lymphocytes were correlated with 21 genera, such as Lactobacillus, Bifidobacterium, and several genera of Lachnospiraceae (Figure 5A). CD25 positive lymphocytes were associated with 7 genera, including Bilophila, Lachnospiraceae_UCG_001, and Rumimiclostridium. Therefore, the Treg-associated bacteria involved the intersection of CD4 and CD25 positive lymphocytes-associated bacteria, such as Bilophila, Lachnospiraceae_UCG_001, and Rumimiclostridium, as well as Lactobacillus and Candidatus. Then the relationship between the human intestinal bacteria that showed significant changes in contents after WBV and body characteristics was also explored by Spearman correlation analysis. The results demonstrated that *Eubacterium coprostanoligenes* was related to weight and height, while *Pediococcus acidilactici* was associated with BMI and weight; and *Lactobacillus paraplantarum* was related to age (Figure 5B).

Next, Spearman correlation analysis was performed toward the interrelationship between the genera with high abundance within the mice and human microbiota, the cut-off value > 0.8 and *p* < 0.05 were applied to filter the data, respectively. The constructed correlation network of mice fecal microbiota showed that the interactions between the bacteria significantly changed in response to WBV (Figure 5C). The same phenomenon also occurred in human fecal microbiota, and the interaction between bacteria changed on the 10th and 30th day of the vibration (Figure 5D and Supplementary Figure 13). The above results suggest that WBV induced variation in regulatory T cell differentiation was correlated to changes in intestinal microbiota composition.

## Discussion

WBV has recently been included in the hospital's patient rehabilitation training catalog, which may play a significant role in preventing osteoporosis and losing weight (Reijne et al., 2016; Swe et al., 2016; McMillan et al., 2017), stimulating the secretion of growth hormone (GH) and testosterone in male students (Cardinale et al., 2010). Recently, some researchers reported that appropriate vibration training was a benefit to metabolic disorders such as obesity and diabetes (Yin et al., 2015; Zhang et al., 2016). In this study, the mice and human volunteers were placed on the vibration instrument for 30 min vibration every day for more than 30 days, and the immunological results in mice showed that WBV alters regulatory T cell differentiation. Since the differentiation in immune cells in the body are usually associated with changes in the human microbiota, therefore the possible changes of microbiota after WBV treatment were characterized in mice and human body. The microbiome results revealed that WBV affected the intestinal microbiome and makes the difference between individuals greater. Interestingly, it appeared that *Lactobacillus* spp. were sensitive to WBV stimulation, the content of *Lactobacillus animalis* in the intestine of mice was significantly increased in response to vibration, while the contents of *Lactobacillus paraplantarum*, and *Lactobacillus sanfranciscensis* in the human body were significantly changed concomitant with WBV. The difference of Lactobacillus variation occurred in the mouse and the human intestines in response to WBV may be due to the distinction in the composition of the two microbiotas, and their intestinal microenvironment is completely divergent. Finally, the correlation analysis results revealed that WBV induced changes in intestinal microbiota composition, such as *Lactobacillus* spp., were correlated to the regulatory T cell differentiation in mice and physical characteristics in human. This study implies that WBV has potential interventional effects on microbiota and immune-related diseases, so WBV-induced changes in microbiota, immune state and inflammation of the body deserve further validation and investigation in the future.

The intestinal microbiota has a profound influence on metabolism, tissue development, and homeostasis of the intestinal immune system. Components of microbiota have been shown to initiate inflammation and also regulate immune cells (Nicholson et al., 2012). A variety of mechanisms have been proposed between the gut microbes and immune response. For example, the alteration of the gut microbiome may promote the intestinal permeability, change butyrate, and lipopolysaccharide (LPS) productions, while butyrate and LPS level could modulate the immune response and inflammation (Noverr and Huffnagle, 2004). Besides, the changes of the gut microbiota could alter the energy homeostasis, modulate intestinal barrier integrity, change the gastrointestinal peptide hormone secretion, promote fat accumulation, and modulate host inflammatory status (Amyot et al., 2012).

Previous studies reported that Lactobacilli down-modulated the maturation of DCs and induced the *in vitro* expansion of CD4 positive T cells, which resemble regulatory T cells (von der Weid et al., 2001; Christensen et al., 2002). Lactobacillus is a Gram-positive, facultative anaerobic or microaerobic, rod-shaped, non-spore forming bacteria. They are the main part of the lactic acid bacteria family (i.e., they convert sugar to lactic acid). Members of the genus Lactobacillus have long been considered to be one of the most abundant microorganisms in the human gastrointestinal (GI) tract and are associated with good intestinal health (Heeney et al., 2018). Animal studies have shown that Lactobacilli have a wide range of roles in the prevention and treatment of infectious diseases, the reduced Interleukin-1β-mediated inflammation and improved barrier function demonstrated the potential of lactobacilli to prevent or reverse intestinal damage during infection (Hirao et al., 2014). A report investigating the fecal microbiome of IBS patients and healthy subjects concluded that lactobacillus was depleted in patients with IBS who were predominantly diarrhea (Liu et al., 2017). The changes in microbiota and immunity triggered by WBV are in constant process of mutual shaping. Previous studies have found that certain components of the microbiota specifically affect the accumulation and activity of Treg cells, among which Lactobacilli and Bifidobacteria are involved in the induction of Treg cells (Tanoue and Honda, 2012). In the intestine, one of the most important effectors produced by Treg cells is IL-10, which is indispensable for the maintenance of intestinal immune homeostasis and micro-ecological balance. Given that autochthonous Lactobacillus plays a crucial role in the resolution of infectious disease and recovery of immune homeostasis, the correlation between WBV-induced content change in *Lactobacillus* spp. and T cell differentiation and clinical parameters might be related to disease occurrence and deserves in-depth study. A report demonstrated that vertical vibration could alleviate the severity of symptoms in patients with chronic functional constipation (Wu et al., 2012), verified the therapeutic effect of WBV. In addition, inflammation-related diseases in the body may be alleviated, although sometimes the symptoms may be less pronounced. Our research will provide the basis for future non-invasive treatment of microbial and immune related diseases.

## Data Availability Statement

The sequencing data were uploaded to the NCBI website with the BioProject ID PRJNA561665 and PRJNA561686 available at https://www.ncbi.nlm.nih.gov/sra/PRJNA561665 or https://www.ncbi.nlm.nih.gov/sra/PRJNA561686.

## Ethics Statement

The studies involving human participants were reviewed and approved by the ethic committee of Binzhou Medical University Hospital (Binzhou, Shandong Province, China). The patients/participants provided their written informed consent to participate in this study. The animal study was reviewed and approved by the ethic committee of School of Basic Medical Science, Shandong University (Jinan, Shandong Province, China).

## Author Contributions

NS and MX collected the mice samples and prepared the immunological results. XLi collected the human samples and clinical characteristics of the patients. QF, XLa, and ML analyzed the sequencing data and prepared the related figures. SL designed the research and prepared the manuscript and modified the figures. RL and CL edited the manuscript. TD organized the figures. DW assisted SL in designing the research and enrolling volunteers. All authors have reviewed the final version of the manuscript and approved this submission.

### Conflict of Interest

The authors declare that the research was conducted in the absence of any commercial or financial relationships that could be construed as a potential conflict of interest.
