# Supplementary material for: Whole Body Vibration Triggers a Change in the Mutual Shaping State of Intestinal Microbiota and Body's Immunity
Source: Front Bioeng Biotechnol. 2019 Nov 29;7:377. doi: 10.3389/fbioe.2019.00377 (PMC6895539; doi:10.3389/fbioe.2019.00377)
Supplement: Supplementary file 1 [file Table_1.DOC]

Supporting information

Supplementary Table 1

| Number | Age | Gender | Height | Weight | BMI |
| --- | --- | --- | --- | --- | --- |
| 11 | 22~37 | 9 M,2 F | 164~180 cm | 53~96 kg | 17.99~31.71 |

Supplementary Table 1. Information of the human volunteers.

Supplementary Table 2

| Samples | Raw PE | Combined | Qualified | Base(nt) | AvgLen(nt) | GC% | Effective% |
| --- | --- | --- | --- | --- | --- | --- | --- |
| Control 1 | 62,693 | 57,624 | 51,198 | 20,651,896 | 414 | 54.21 | 79.62 |
| Control 2 | 65,890 | 59,773 | 53,132 | 21,617,206 | 415 | 54.26 | 79.12 |
| Control 3 | 79,636 | 72,186 | 64,016 | 25,829,000 | 413 | 54.33 | 78.61 |
| Control 4 | 65,371 | 59,265 | 52,159 | 21,565,780 | 419 | 53.59 | 78.73 |
| Control 5 | 69,391 | 63,471 | 56,356 | 22,864,012 | 412 | 54.03 | 79.89 |
| Treatment 1 | 61,795 | 56,401 | 50,046 | 20,486,488 | 417 | 53.75 | 79.41 |
| Treatment 2 | 73,103 | 65,752 | 58,266 | 23,739,631 | 415 | 54.6 | 78.26 |
| Treatment 3 | 79,126 | 71,796 | 63,009 | 26,138,428 | 421 | 54.15 | 78.38 |
| Treatment 4 | 75,349 | 69,368 | 61,164 | 25,217,255 | 420 | 53.48 | 79.68 |
| Treatment 5 | 66,763 | 61,504 | 54,434 | 22,411,625 | 421 | 54.56 | 79.77 |

Supplementary Table 2. Assessment of the sequencing data quality.

Supplementary Figure 1


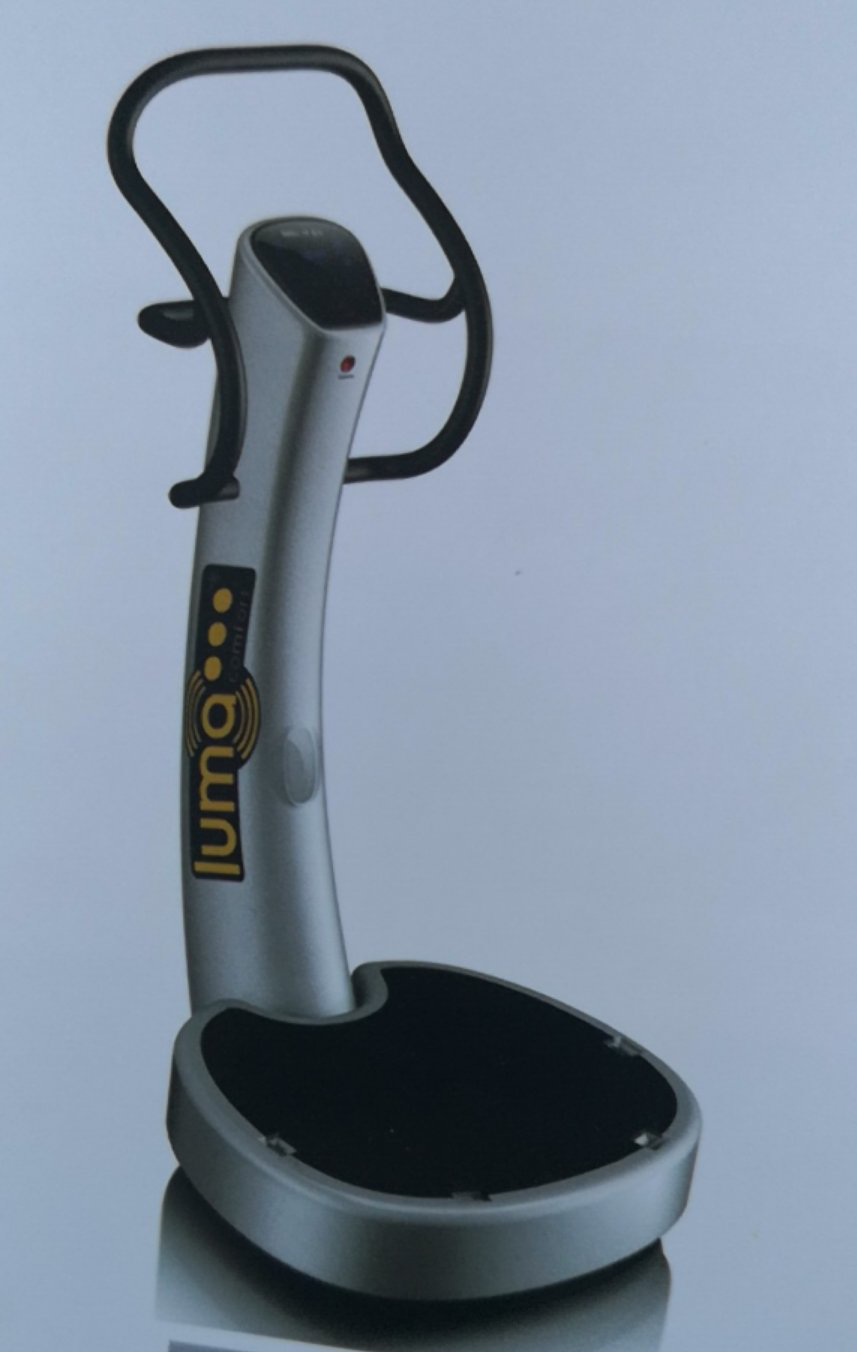


Supplementary Figure 1. The vibration instrument used in the experiment.

Supplementary Figure 2

Supplementary Figure 3. The average daily food intake of mice.

Supplementary Figure 3

Supplementary Figure 3. The curve of the amount of water consumed by the mice per day over time.

Supplementary Figure 4


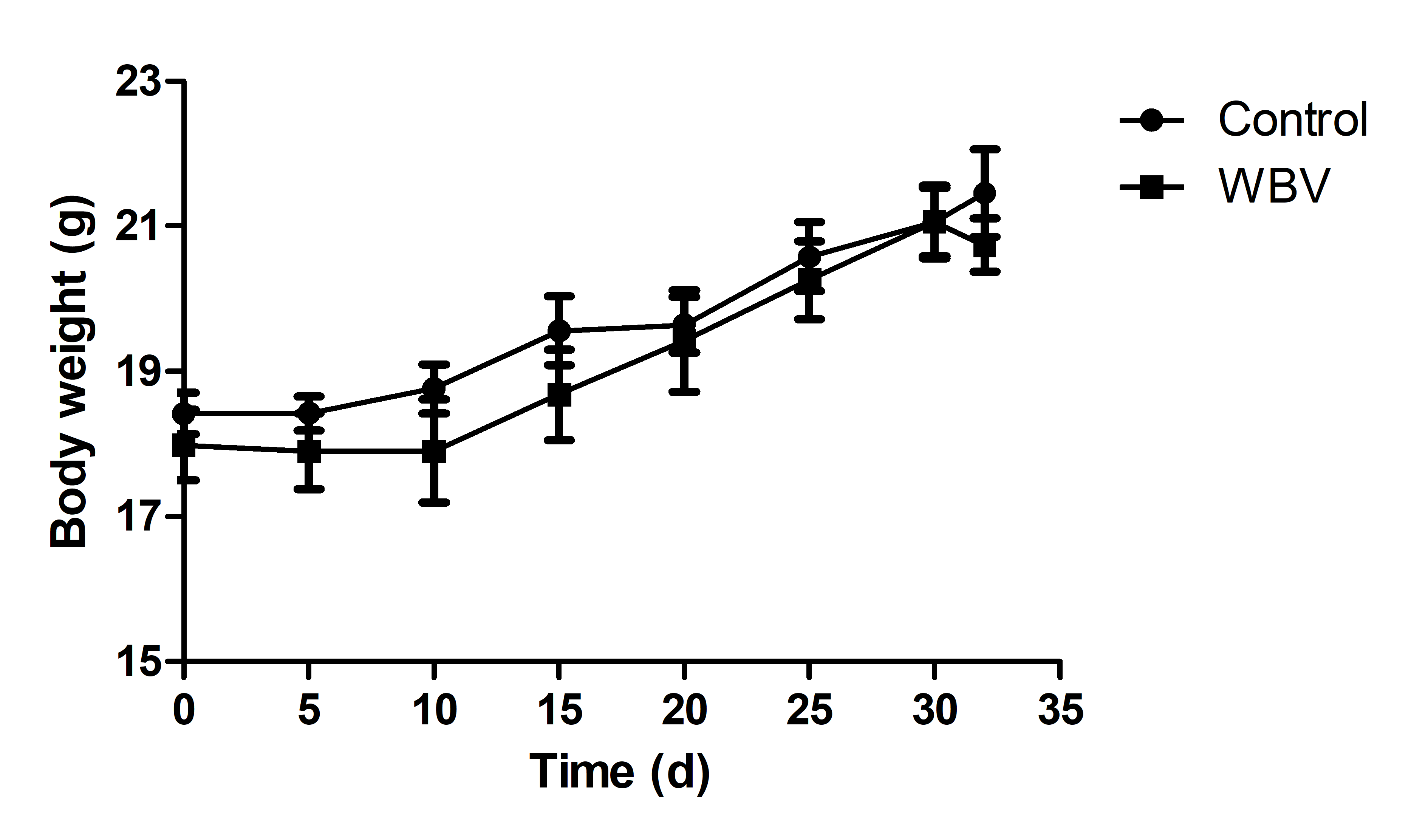


Supplementary Figure 4. Changes in mouse body weight over time.

Supplementary Figure 5

Supplementary Figure 5. Statistics of sample OTUs number.

Supplementary Figure 6


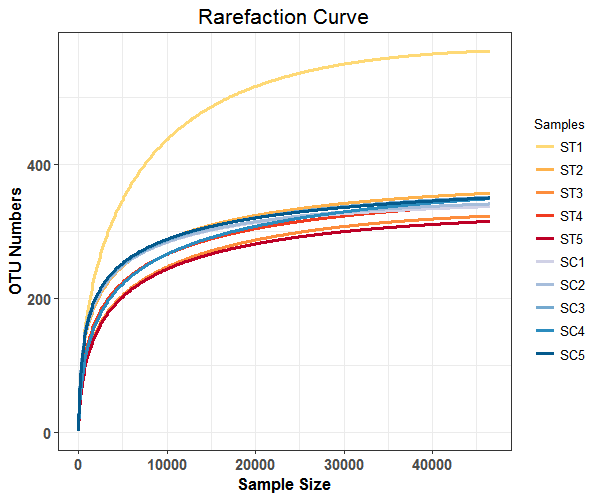


Supplementary Figure 6. Rarefaction curve: The abscissa is the number of randomly selected sequences and the ordinate is the number of OTUs clustered based on the number of sequences. Each curve represents a sample, and the curve tends to be stable, indicating that the number of extracted sequences is enough to reflect the OTUs of the sample.

Supplementary Figure 7


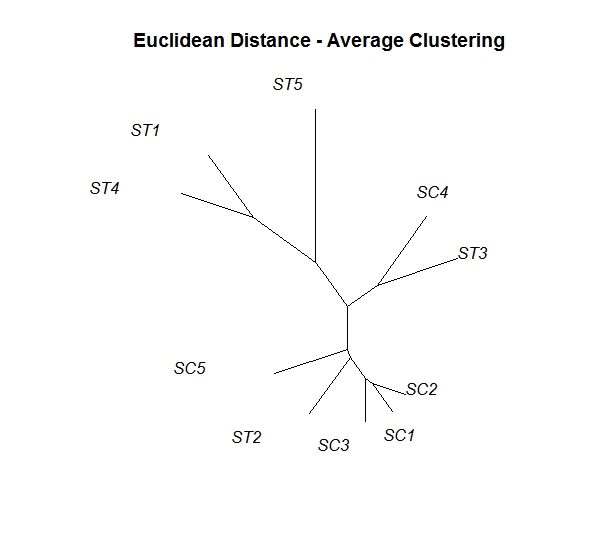


Supplementary Figure 7. Clustering analysis result of all the samples, and samples were clustered together with various Euclidean distance.

Supplementary Figure 8


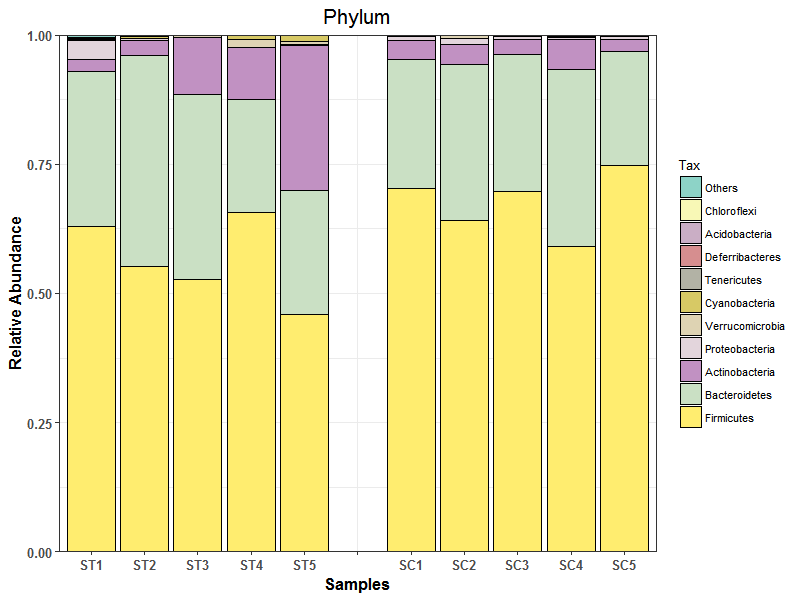


Supplementary Figure 8. The intestinal microbial composition of the mice at the phylum level.

Supplementary Figure 9


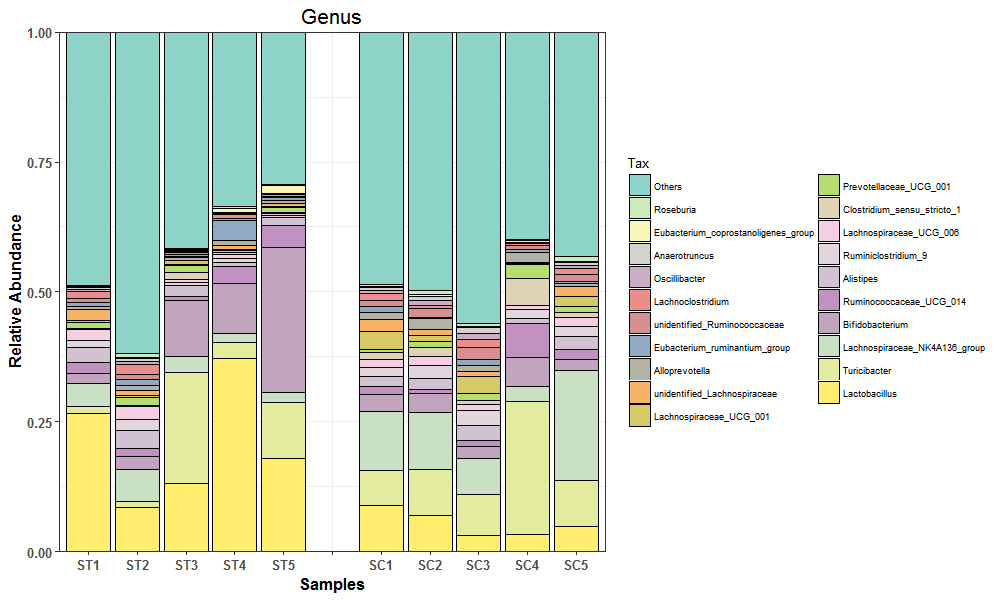


Supplementary Figure 9. The intestinal microbial composition of the mice at the generic level.

Supplementary Figure 10


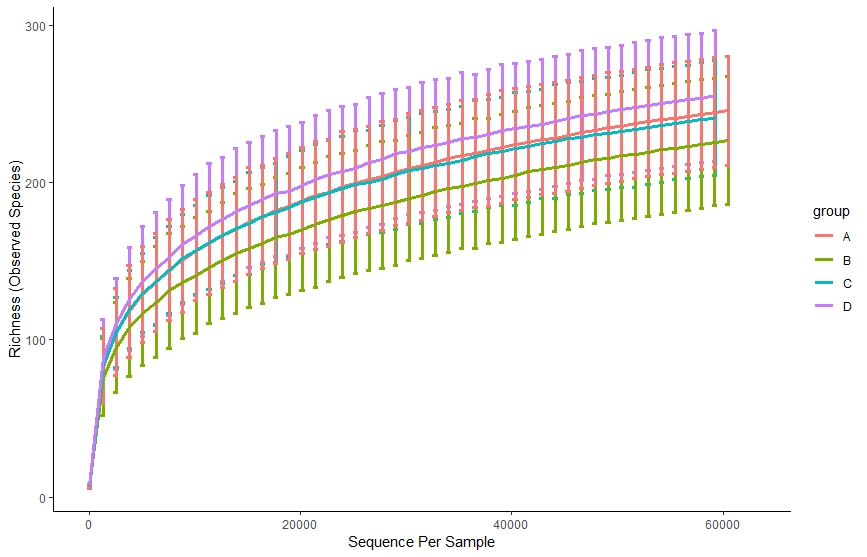


Supplementary Figure 10. Rarefaction curve: The abscissa is the number of randomly selected sequences and the ordinate is the number of observed species based on the number of sequences. Each curve represents a time point, and the curve tends to be stable, indicating that the number of extracted sequences is enough to reflect the observed species of the time point.

Supplementary Figure 11


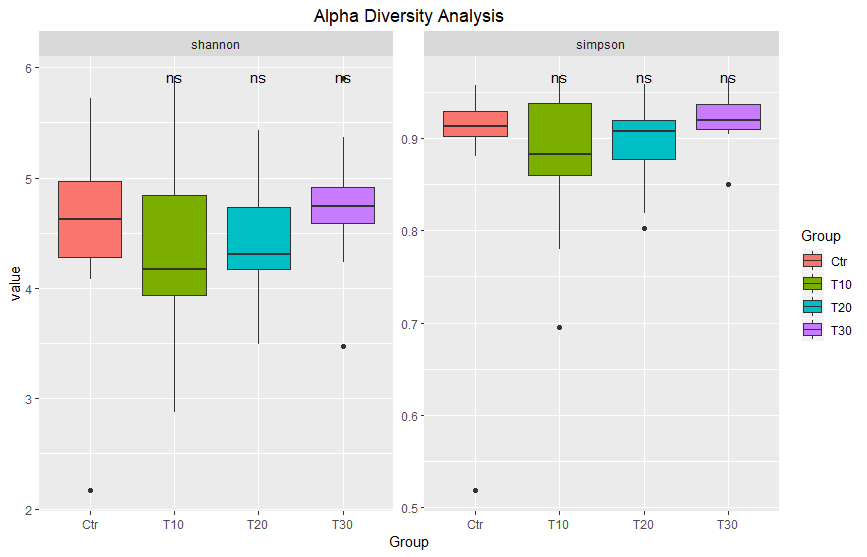


Supplementary Figure 11. Comparison of α-diversity of human fecal flora.

Supplementary Figure 12


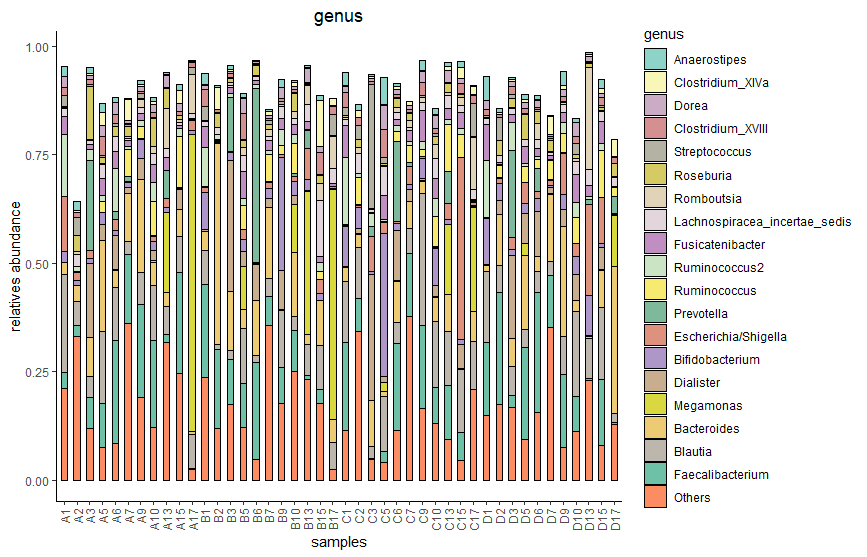


Supplementary Figure 12. The intestinal microbial composition of the human at the generic level.

Supplementary Figure 13


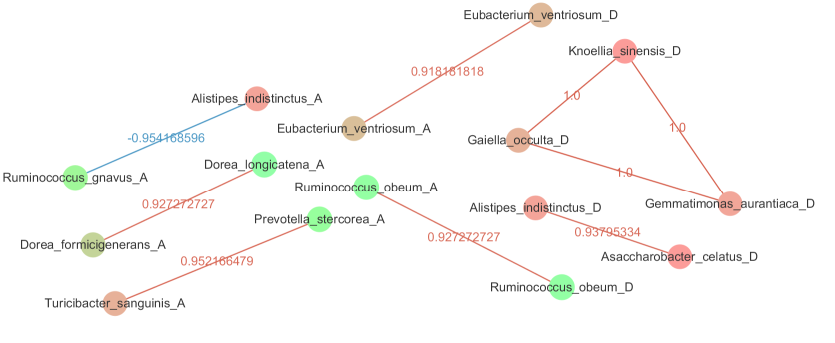


Ctrl T30

Supplementary Figure 13. Interrelationship between the genera with high abundance within the human microbiota.
